# Celiac Disease-Related Inflammation Is Marked by Reduction of Nkp44/Nkp46-Double Positive Natural Killer Cells

**DOI:** 10.1371/journal.pone.0155103

**Published:** 2016-05-12

**Authors:** Irene Marafini, Ivan Monteleone, Davide Di Fusco, Silvia Sedda, Maria Laura Cupi, Daniele Fina, Alessandro Omero Paoluzi, Francesco Pallone, Giovanni Monteleone

**Affiliations:** Department of Systems Medicine, University of Rome “Tor Vergata”, Rome, Italy; Harvard Medical School, UNITED STATES

## Abstract

**Introduction and Aim:**

Natural killer (NK) cells are a first line of defence against viruses and down-regulation of NK cell cytotoxic receptors represents one of the strategies by which viruses escape the host’s immune system. Since onset of celiac disease (CD), a gluten-driven enteropathy, has been associated with viral infections, we examined whether CD-associated inflammation is characterized by abnormal distribution of NK cell receptors involved in recognition of viral-infected cells.

**Materials and Methods:**

Intraepithelial mononuclear cells, isolated from duodenal biopsies of active and inactive CD patients and healthy controls (CTR) and jejunal specimens of obese subjects undergoing gastro-intestinal bypass, were analysed for NK cell markers by flow-cytometry. Expression of granzyme B, interleukin (IL)-22 and tumor necrosis factor (TNF)-α was as assessed in freshly isolated and toll-like receptor (TLR) ligand-stimulated cells.

**Results:**

The percentages of total NK cells and NKT cells did not significantly differ between CD patients and CTR. In active CD, the fractions of NKp30+ NK cells, NKG2D+ NK cells and NKG2D+ NKT cells were significantly increased as compared to inactive CD patients and CTR. In contrast, CD-associated inflammation was marked by diminished presence of NKG2A+ NK cells and NKG2A+ NKT cells. The fractions of NK cells and NKT cells expressing either NKp44 or NKp46 did not differ between CD and controls, but in CD less NK cells and NKT cells co-expressed these receptors. NKp44/NKp46-double positive cells produced granzyme B and IL-22 but not TNF-α and responded to TLR ligands with enhanced expression of granzyme B.

**Conclusions:**

These data indicate that active phase of CD associates with reduced presence of NKp44/NKp46-double positive NK cells and NKT cells in the epithelial compartment.

## Introduction

Natural killer (NK) cells belong to the large family of innate lymphoid cells and are an evolutionary conserved innate asset of the immune system to fight infections and tumour growth [1]. NK cells produce a vast array of pro-inflammatory cytokines and cytotoxic products, such as granzyme B and perforin, thus contributing to the lysis of target cells [2]. The cytolytic function of NK cells is regulated by the expression of surface receptors, the so-called NK cell receptors that either block or enhance the NK-mediated cytotoxicity [2, 3]. In particular, under physiologic conditions, target cells are protected from NK-mediated cytotoxicity by the expression of HLA class I molecules [4]. NK cells express on their cell surface HLA-specific inhibitory receptors (i.e. CD94/NKG2A heterodimers), which interact with the ligands on normal target cells and inhibit NK-mediated cytolytic activity [4]. The absence of these inhibitory interactions renders target cells susceptible to NK-mediated cytotoxicity [5]. Induction of cytotoxicity is mediated by non-HLA-specific activating NK receptors (i.e. NKp30, NKp44, and NKp46). There is a strict correlation between surface density of activating NK receptors and NK-mediated cytotoxicity against target cells [6]. Indeed, NK cells expressing low NK cell receptor surface density are poorly or even non cytolytic against most target cells [6]. Another activating NK cell receptor is NKG2D, which, unlike NKp30, NKp44, and NKp46, is also expressed by virtually all cytolytic T lymphocytes. In NK cells, NKG2D expression does not necessarily correlate with that of NKp30, NKp44, and NKp46[7] [8]. The whole repertoire of specific ligands of activating NK cell receptors on normal, virus-infected and tumoral cells is not yet known, though the ligands for NKG2D include the MICA and MICB stress-inducible molecules and the ULBP (UL16-binding protein) major histocompatibility complex class I–related molecules [9].

One of the strategies used by microbes to escape the surveillance of the immune system is the down-regulation of activating NK cell receptors. For example, carriers of herpes virus 8 have a substantial alteration of NK cell receptor repertoire with reduced expression of NKp46, NKp30 and NKG2D that contribute to maintain viral latency and to promote in the later stages the growth of Kaposi sarcoma [10]. Cytokines produced in response to human cytomegalovirus infections significantly reduce NKG2D expression on NK cells [11] and in HIV-1-infected patients there is a decreased surface densities of NKp30, NKp44, and NKp46, which is associated with defective cytotoxic activity [12].

In celiac disease (CD), a chronic enteropathy triggered by the ingestion of gluten, a persistent and exaggerated mucosal immune response promotes tissue damage [13]. T cells and NK cells infiltrating the epithelial compartment of CD duodenum bear NK receptors that bind specific ligands expressed on enterocytes, thus inducing epithelial injury [14, 15]. Both genetic and environmental factors are supposed to contribute to CD pathogenesis, even though an exclusive causative agent has not yet been identified [16]. A long-standing hypothesis, mainly supported by epidemiological observations, suggests the potential contribution of viral infections in the initiation and/or perpetuation of the tissue destructive inflammatory response in CD. In a Swedish cohort of children who developed CD, one of the main risk factors was exposure to neonatal infections [17]. Another study reported the early onset of CD associated with serological positivity for rotavirus infection [18]. In line with this is the demonstration that, in CD, mucosal inflammation associates with high production of interferon (IFN)-α [19], a cytokine over-produced by virus-infected cells, and activation of intestinal T cells with IFN-α causes CD-like pathology in fetal gut explants [20]. Moreover, early onset of CD has been documented in patients receiving IFN-α for hepatitis or leukaemia [19, 21]. Based upon these observations, it is conceivable that defects in the expression of activating NK cell receptors impair the ability of NK cells to kill virus-infected cells thus contributing to trigger/amplify local inflammation. To begin to address this issue, we examined whether CD-associated inflammation is characterized by abnormal distribution of NK cell receptors recognizing virus-infected cells.

## Materials and Methods

### Patients and samples

Duodenal biopsies were taken from 16 patients with active CD (ACD) at the time of diagnosis, 10 patients with inactive CD on a gluten-free diet and 16 normal controls. All patients with ACD were on a gluten-containing diet, were positive for both IgA anti-endomysium (EMA) and IgA anti-tissue transglutaminase 2 (TG2) and had villous atrophy on histological examination. ICD patients were on a gluten-free diet for at least 2 years, were EMA and anti-TG2 negative and none of them had villous atrophy on histological examination. Control group included duodenal biopsies of patients who underwent upper endoscopy for gastrointestinal symptoms, had no macroscopic/microscopic alteration, and were EMA and anti-TG2 negative. Additional control samples included normal jejunal specimens of subjects undergoing gastro-intestinal bypass for obesity. All patients and controls had no clinical or laboratory sign of viral infections. Each patient who took part in the study gave written informed consent and the independent local Ethics Committee of the University hospital of Tor Vergata approved the study protocol.

### Cell isolation and culture

Human intra-epithelial cells were isolated as previously described with minor modifications [22]. Briefly, biopsies taken from controls, ICD patients and ACD patients and surgical specimens of normal subjects were freed of mucus and epithelial cells in sequential steps with dithiothreitol and ethylene diminetetracetic acid (EDTA) in order to obtaining a suspension of intra-epithelial cells. These cell preparations were resuspended (1x10^6^/ml) in RPMI-1640 supplemented with 10% fetal bovine serum, penicillin (100μg/ml), streptomycin (100μg/ml), and gentamycin (50μg/ml; Lonza, Milan, Italy). Cells were either freshly stained for flow cytometry analysis or cultured with peptidoglycan (PGN, 10μg/ml), poly: IC (5μg/ml), CpG (1μg/ml), LPS (100ng/ml) for 24 hours and then analyzed by flow cytometry. Phorbolmyristate acetate (PMA, 10 ng/ml), ionomycin (1 mg/ml), and brefeldin A (10 mg/ml; eBioscience, San Diego, CA) were added to the cultures in the last 5 hours in order to evaluate cytokine production. Peripheral blood mononuclear cells (PBMC) were isolated from EDTA-stabilized peripheral blood samples of patients and controls by Ficoll gradients and analyzed for the frequency of NK cells by flow-cytometry.

### Flow cytometry

Cells were immunostained with the following monoclonal anti-human antibodies: APC-H7 anti-CD45 (clone 2D1), V450 anti-CD56 (clone B159), Percp anti-CD3 (clone SK7), PE anti-CD314 (NKG2D; clone 1D11), PECy7 anti-NKp46 (clone 9E2/Nkp46), AlexaFuor 647 anti-NKp44 (clone p44-8), PE anti-CD337 (NKp30; clone p30-15), PE anti-tumor necrosis factor (TNF)-α (clone Mab11), (all from Becton Dickinson, Milan, Italy), PE-anti Granzyme B (clone GB12; Invitrogen), PE anti-interleukin (IL)-22 (clone 22URTI), FITC anti-CD103 (clone B-Ly7) (eBioscience) and Percp anti-NKG2A (clone 131411; R&D Systems). In all experiments, appropriate isotype control IgGs (Becton Dickinson and eBioscience) were used. All antibodies were used at 1:100 final dilution. For intracellular immunostaining, cells were fixed and permeabilized using staining buffer set and permeabilization buffer (both from eBioscience) according to the manufacturer’s instruction. Cells were analyzed by flow cytometry (FACSverse, BD Bioscience, San Jose, CA).

### Statistical analysis

Nonparametric methods were used for statistical analysis of the data using the Graph Pad Prism software version 5.00 (Graph Pad software Inc, La Jolla, CA, USA). Differences in the fractions of surface receptors expressed by NK and NKT cells in samples taken from controls, ICD and ACD patients were evaluated using the Mann–Whitney U-test.

## Results

### NK cells infiltrating the epithelial compartment of patients with active celiac disease exhibit high levels of the activating receptors NKp30 and NKG2D and low levels of the inhibitory receptor NKG2A

**Fig 1:**
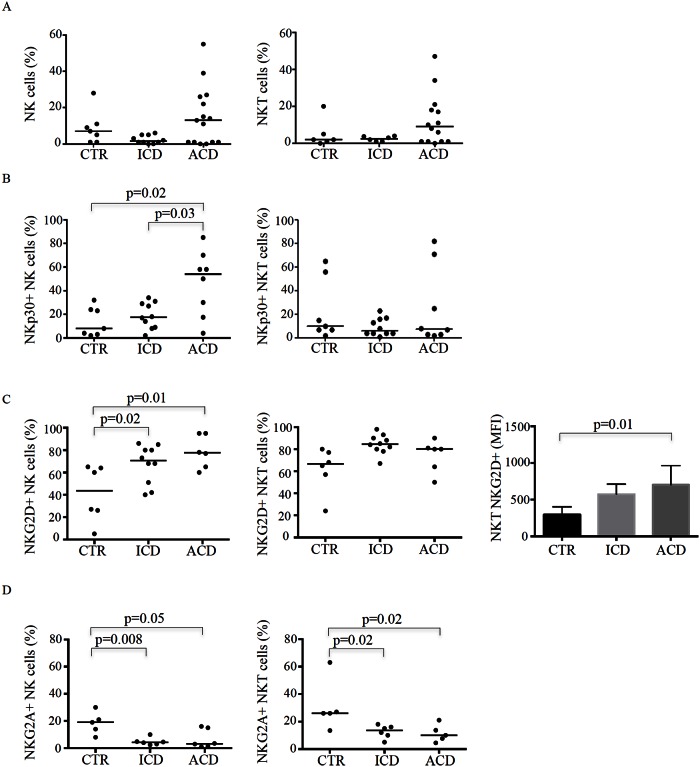
The intestinal epithelial compartment of celiac disease contains subpopulations of NK cells and NKT cells characterized by increased levels of NKp30 and NKG2D and low levels of NKG2A. Intraepithelial mononuclear cells isolated from duodenal biopsies of 7 normal controls (CTR), 10 inactive celiac disease (ICD) patients and 15 active celiac disease (ACD) patients were stained with CD45, CD103, CD3, CD56, NKp30, NKG2D and NKG2A antibodies. Each point in the graph indicates the percentage of positive cells in a single sample of a single patient. The horizontal bars represent the median values. A. Left graphs show the percentages of NK cells (CD45+,CD103+,CD56+) in CTR, ICD patients and ACD patients. Right graph shows the percentages of NKT cells (CD45+,CD103+,CD56+, CD3+) in CTR, ICD patients and ACD patients. B. Left graph shows the percentages of NKp30+ NK cells in CTR, ICD patients and ACD patients. Right graph shows the percentages of NKp30+ NKT cells in CTR, ICD patients and ACD patients. C. Left graph shows the percentages of NKG2D+ NK cells in CTR, ICD patients and ACD patients. Middle graph shows the percentages of NKG2D+ NKT cells in CTR, ICD patients and ACD patients. Right graph shows mean fluorescence intensity (MFI) values for NKG2D+ in NKT cells isolated from CTR, ICD patients and ACD patients. Data indicate mean and standard error. D. Left graph shows the percentages of NKG2A+ NK cells in CTR, ICD patients and ACD patients. Right graph shows the percentages of NKG2A+ NKT cells in CTR, ICD patients and ACD patients.

Initially we analysed the distribution of NK cells and NKT cells in the intraepithelial compartment of the duodenum of patients with ACD, patients with ICD and controls. Intra-epithelial cells were immunostained with fluorescent antibodies and analysed by flow-cytometry. Intraepithelial cells were initially gated on the lympho-monocytic area in the FSC/SSC plot, then on the CD45+ CD103+ population and finally on the CD3+CD56+ population to analyse NKT cells and on CD56+ cells to analyse NK cells (S1 Fig). The fractions of NK cells (CD45+, CD103+, CD56+) and NKT cells (CD45+, CD103+, CD56+, CD3+) did not differ among the three groups (Fig 1A).

Next, we assessed the expression of inhibitory and activating NK cell receptors. To this end, we initially evaluated NKG2A, NKG2D and NKp30 in intraepithelial cell samples by flow-cytometry. The percentage of NKp30+ NK cells was greater in ACD patients than in ICD patients and controls, while the fractions of NKp30-expressing NKT cells did not differ among groups (Fig 1B). NKG2D-expressing NK cells were more abundant in ACD patients and ICD patients compared to normal controls, while the percentages of NKG2D-positive NKT cells did not differ among the three groups. However, assessment of mean fluorescence intensity for NKG2D revealed higher surface density of the receptor in NKT cells isolated from ACD patients compared to normal controls, whereas there was no difference between ACD and ICD (Fig 1C). In contrast, the percentages of NK cells and NKT cells positive for the inhibitory receptor NKG2A were significantly decreased in ACD and ICD compared to normal controls (Fig 1D).

These data indicate that the intestinal epithelial compartment of ACD patients is infiltrated with distinct subpopulations of NK cells, which express high levels of activating receptors and are devoid of the inhibitory NKG2A.

### NKp44/NKp46 double positive NK cells and NKT cells are decreased in active celiac disease

**Fig 2:**
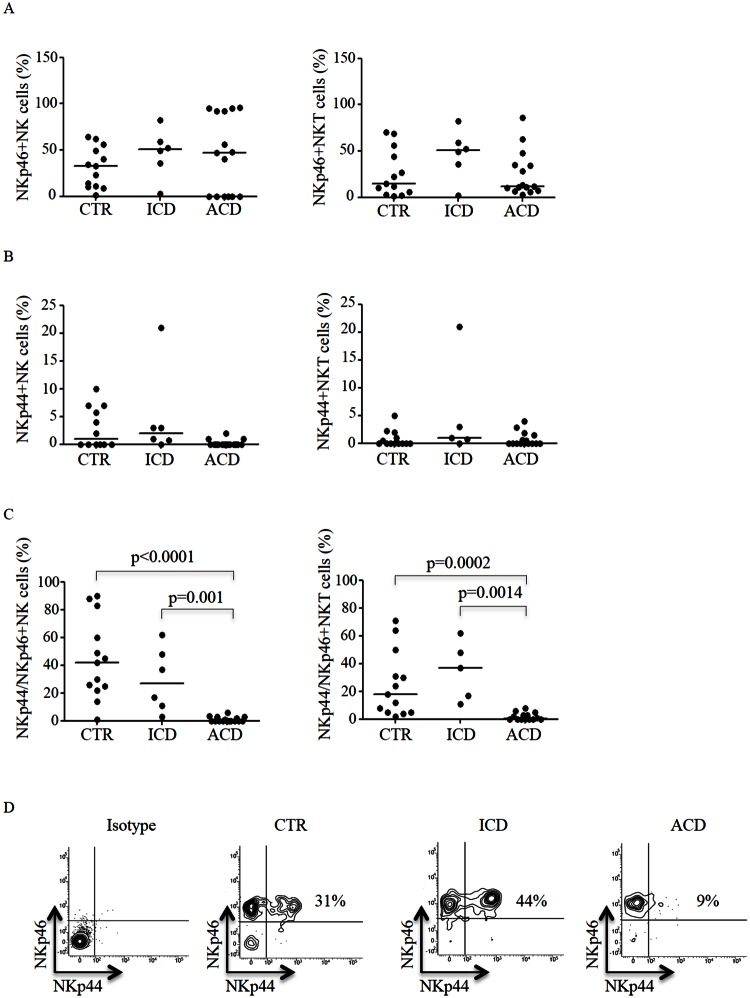
NKp44/NKp46-double positive NK cells and NKT cells are decreased in active celiac disease (ACD). A-C. Intraepithelial mononuclear cells isolated from duodenal biopsies of 13 normal controls (CTR), 6 inactive celiac disease (ICD) patients and 15 ACD patients were stained with CD45, CD103, CD56, CD3, NKp44 and NKp46 antibodies. Panel A shows the percentages of NKp46+ NK (CD45+,CD103+, CD56+; left) cells and NKT (CD45+,CD103+, CD56+, CD3+; right) cells in CTR, ICD patients and ACD patients. Panel B shows the percentages of NKp44+ NK (left) cells and NKT (right) cells in CTR, ICD patients and ACD patients. Panel C shows the percentages of NKp46/NKp44-double positive NK (left) cells and NKT (right) cells in CTR, ICD patients and ACD patients. Each point in the graph indicates the percentage of positive cells in a single sample of a single patient. The horizontal bars represent the median values. D. Representative contour-plots show NKp44 and NKp46 expression in NK cells and NKT cells of CTR, ICD patients and ACD patients.

As pointed out above, NK cells can express additional activating receptors, namely NKp44 and NKp46, which are involved in killing of virus-infected cells [3]. Therefore, the next studies were performed to evaluate the expression of these two receptors in intestinal intraepithelial cells of patients and controls. NK cells and NKT cells expressing either NKp44 or NKp46 were equally distributed among the three groups (Fig 2A and 2B). In contrast, NKp44/NKp46-double positive NK cells and NKT cells were significantly reduced in ACD as compared to ICD and controls (Fig 2C and 2D).

### NKp44/NKp46-double positive cells produce Granzyme B and IL-22 and respond to microbe-derived products

**Fig 3:**
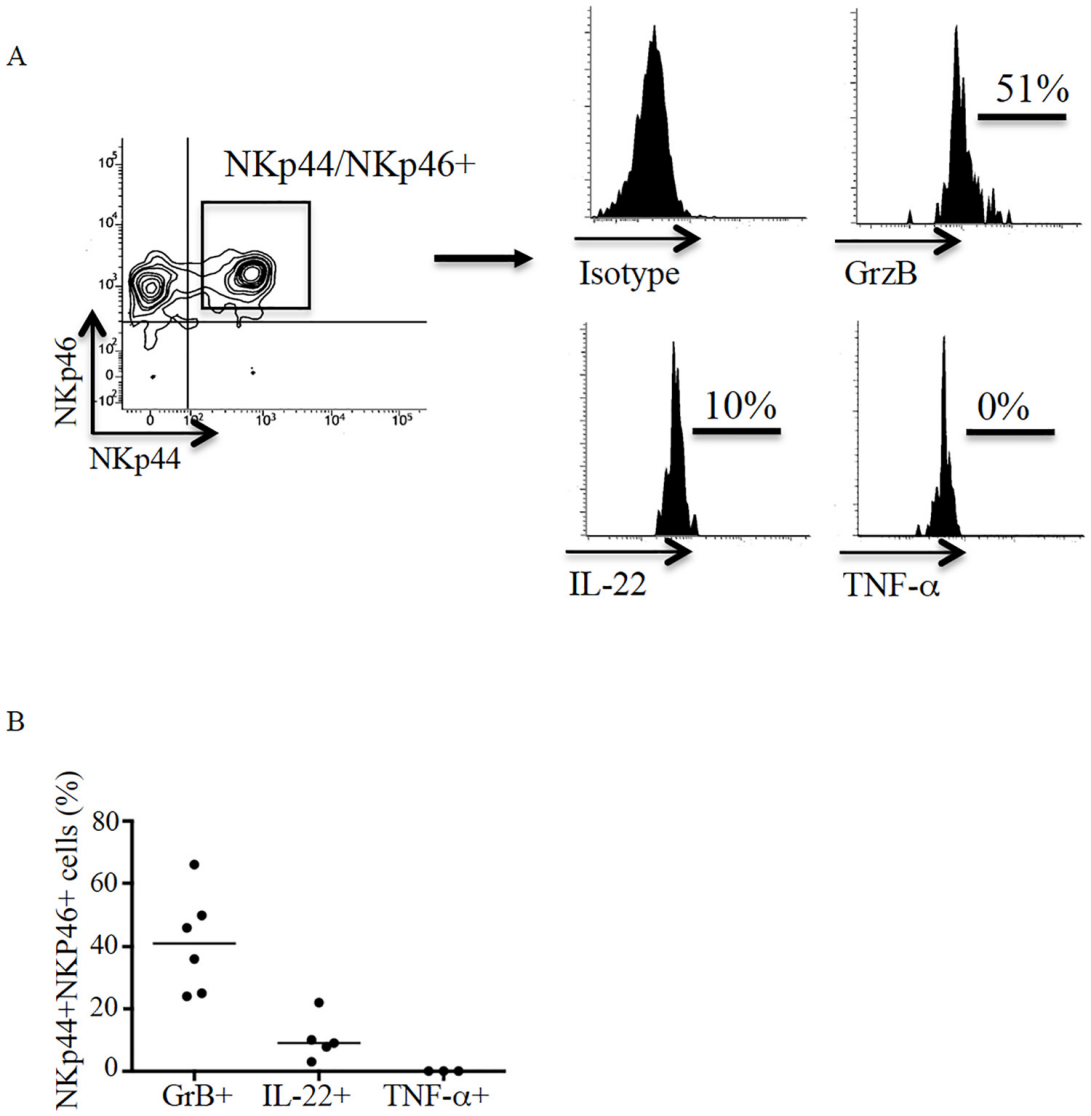
NKp44/NKp46-double positive NK cells and NKT cells are a source of granzyme B and IL-22. Intraepithelial mononuclear cells isolated from duodenal biopsies of normal controls (CTR) were stained with CD45, CD103, CD56, CD3, NKp44, NKp46, granzyme B, IL-22 and TNF-α antibodies. A. Panel A shows representative histogram-plots of granzyme B, IL-22 and TNF-α expression in NKp44/NKp46-double positive NK cells and NKT cells. Data are representative of respectively 6, 5 and 3 separate experiments. B. Panel B shows the percentages of granzyme B, IL-22 and TNF-α expression in NKp46/NKp44-double positive cells. Each point in the graph indicates the percentage of positive cells in a single sample of a single patient.

In subsequent studies NKp44/NKp46-double positive NK cells and NKT cells were evaluated for the expression of granzyme B and cytokines. Intra-epithelial cells were immunostained with fluorescent antibodies for granzyme B, TNF-α and IL-22 and analysed by flow-cytometry. Such an analysis was restricted to control cells, as NKp44/NKp46-double positive cells are virtually absent in ACD, thus making difficult FACS staining. Approximately 50% of NKp44/NKp46-double positive NK cells and NKT cells produced granzyme B and 10% of cells were positive for IL-22 (Fig 3A and 3B). In contrast, CD45+ cells but not NKp44/NKp46-double positive NK cells and NKT expressed TNF-α (Fig 3A and 3B and not shown).

**Fig 4:**
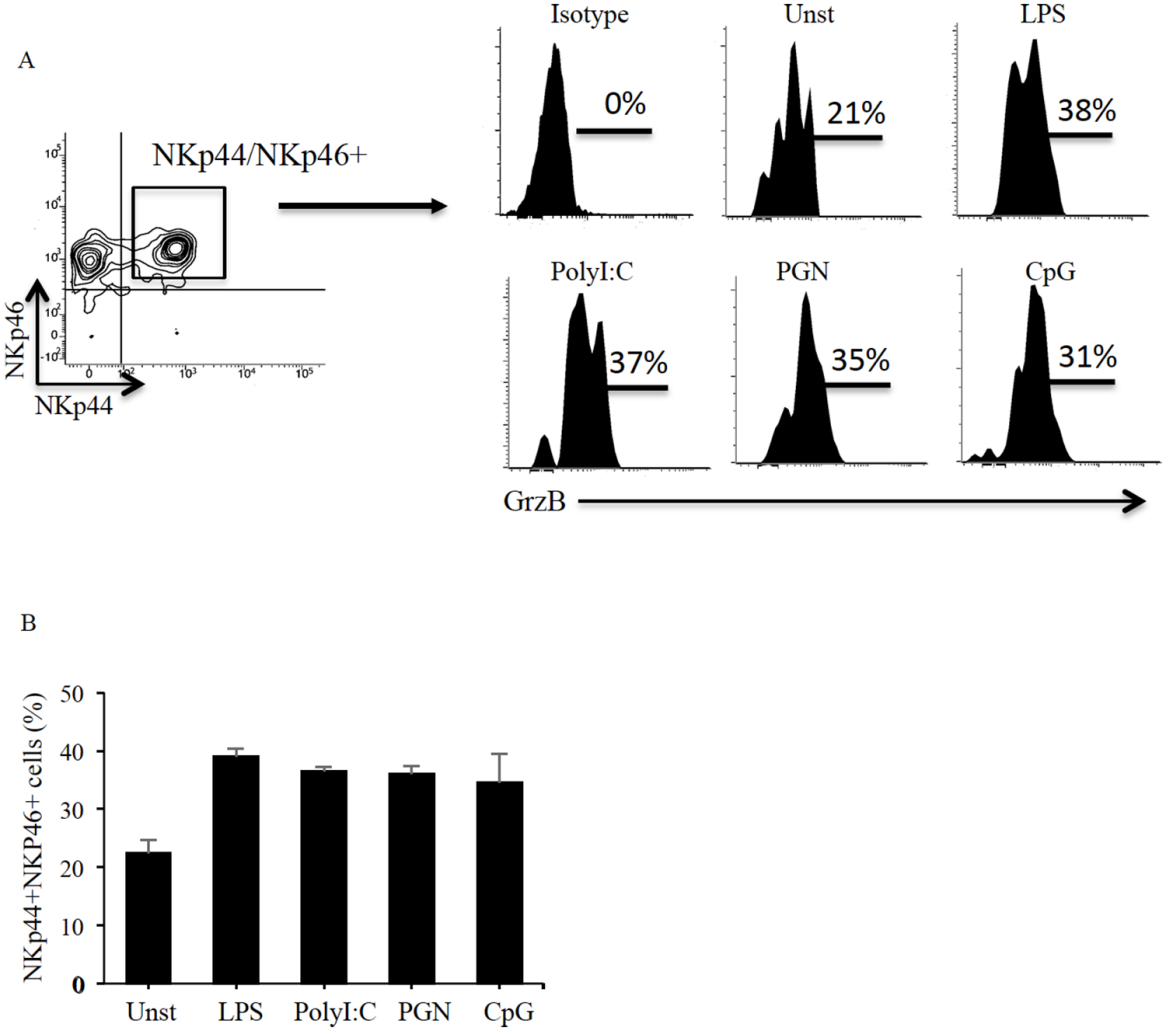
Stimulation of normal intra-epithelial cells with toll-like receptor ligands increases granzyme B expression in NKp44/NKp46-double positive NK cells and NKT cells. Intraepithelial mononuclear cells, isolated from jejunal specimens of 3 controls, were either left unstimulated (Unst) or stimulated with LPS, poly I:C, PGN and CpG. Granzyme B expression was then evaluated in NKp44/NKp46-double positive NK (CD45+,CD103+, CD56+) cells or NKT (CD45+,CD103+, CD56+, CD3+) cells. A. Panel A shows representative histogram-plots of Granzyme B expression in intraepithelial NK and NKT cells either left unstimulated (Unst) or stimulated with LPS, poly I:C, PGN and CpG. B. Panel B shows the percentages of Granzyme B expression in intraepithelial NK and NKT cells either left unstimulated (Unst) or stimulated with LPS, poly I:C, PGN and CpG. Data are representative of 2 different experiments and show an increased expression of Granzyme B, that however do not reach statistical significance.

Since NK cells are an innate source of cytokines during infections, we assessed the response of NKp44/NKp46-double positive cells to TLR ligands. The number of intraepithelial cells that can be isolated from pinch biopsy samples is not sufficient to carry out functional studies. Therefore, the subsequent studies were performed using intra-epithelial cells isolated from jejunal specimens and cultured in the presence of PGN, poly:IC, LPS and CpG, the ligands of TLR2, TLR3, TLR4 and TLR9, respectively. Cells isolated from jejunal specimens and duodenal biopsies were phenotypically similar. After 24 hours, cells were immunostained and production of cytokines by NKp44/NKp46-double positive cells was evaluated by flow-cytometry. Stimulation of cells with poly I:C, PGN, CpG and LPS enhanced expression of granzyme B, but not of TNF-α or IL-22, in NK cells and NKT cells (Fig 4A and 4B and not shown).

## Discussion

CD is a gluten-driven T cell-mediated enteropathy characterized by various degrees of epithelial injury and malabsorption [23]. The exact sequence of events that leads to CD-associated tissue damage is not fully understood, though cytotoxic lymphocytes are supposed to make a major contribution to the epithelial cell death [24]. This process is favored by induction of activating NK receptors on intraepithelial lymphocytes [14]. In particular, during the active phases of the disease, induction of NKG2C on intraepithelial cytotoxic lymphocytes could favor lysis of HLA-E-expressing enterocytes [14, 25]. Our study confirms and expands on such data by showing that high numbers of NK cells and NKT cells expressing NKp30 or NKG2D and reduced numbers of cells expressing the negative receptor NKG2A mark CD-associated mucosal inflammation. We also evaluated the expression of NKp44 and NKp46, two other activating NK receptors, and showed no significant difference between CD and controls in terms of cells expressing either NKp44 or NKp46, while NKp44/NKp46-double positive NK cells and NKT cells were virtually absent in ACD. Meresse and co-workers showed up-regulation of NKp44, but not NKp46, in NKG2C-positive T cells of CD patients [14]. This apparent discrepancy could rely on the different populations evaluated for the expression of NKp44/NKp46, as unlike Meresse and co-workers, our analysis was performed on total intraepithelial cells and not on NKG2C-positive T cells.

NKp44/NKp46-double positive NK cells and NKT cells produced constitutively granzyme B and responded to TLR ligands with enhanced expression of granzyme B, thus suggesting a role for these cells during microbial infections. NK cells bear TLRs and are therefore theoretically able to respond to TLR ligands [26]. However, we were not able to purify a sufficient number of NK cells from intraepithelial mononuclear cell preparations to carry out functional studies. Therefore, our data do not rule out the possibility that the inducing effect of TLR ligands on granzyme B production could be indirect and mediated by other TLR-expressing cell types contained in the intraepithelial cell suspension. Unfortunately, we were not able to purify sufficient numbers of NK and NKT cells from small endoscopic biopsies to carry out functional studies. Therefore, our results are descriptive and the pathogenic relevance of the diminished presence of NKp44/NKp46-double positive NK cells and NKT cells in CD remains unknown. However, a similar finding was documented in patients with chronic viral infections [10–12] and supposed to represent a mechanism by which viruses escape the host’ surveillance. Therefore, one can speculate that deficiency of NKp44/NKp46-double positive NK cells and NKT cells could contribute to the amplification and perpetuation of virus-driven mucosal immune responses, which ultimately cause tissue damage. Viral infections are not the cause of CD, but accumulating evidence suggests a possible role of infectious co-factors in CD development. For instance, epidemiological studies have linked viral infections with onset of CD and experimental work has shown that the active phase of CD is marked by induction of genes associated with viral infections [19]. NKp44/NKp46-double positive NK cells and NKT cells produce also IL-22, a cytokine that stimulates epithelial cell proliferation, defensin and mucous production [27, 28]. Deficiency of such cells could thus interfere with activation of further mechanisms by which the host controls microbial infections.

In our patients, exclusion of gluten from diet reverted histological signs of CD and associated with recovery of NKp44/NKp46-double positive NK cells and NKT cells. Taken together these findings indicate that the lack of NKp44/NKp46-double positive NK cells and NKT cells is not a primary event in CD pathogenesis and suggest a possible role for gluten-driven inflammation in the reduction of such NK cell subpopulation. Further studies in patients with potential CD in whom serological abnormalities associate with no histological alterations will help ascertain whether such a defect precedes the onset of mucosal damage.

In conclusion, this is the first to show that the active phase of CD is characterized by defective mucosal presence of a subset of NK cells, namely NKp44/NKp46-double positive cells producing granzyme B and IL-22, which are known to be involved in the hosts’ response against viruses.

## Supporting Information

S1 Fig Gating strategy for representative dot plots of NK and NKT cells. Intraepithelial cells were gated on the lymphocytic area in the FSC/SSC plot, then on the CD45+ CD103+ population and finally on the CD3+CD56+ (NKT) cell and CD56+CD3- (NK) cell populations.
(DOCX)
