# Supplementary material for: Celiac Disease-Related Inflammation Is Marked by Reduction of Nkp44/Nkp46-Double Positive Natural Killer Cells
Source: PLoS One. 2016 May 12;11(5):e0155103. doi: 10.1371/journal.pone.0155103 (PMC4865226; doi:10.1371/journal.pone.0155103)
Supplement: S1 Fig — Intraepithelial cells were gated on the lymphocytic area in the FSC/SSC plot, then on the CD45+ CD103+ population and finally on the CD3+CD56+ (NKT) cell and CD56+CD3- (NK) cell populations. (DOCX) [file pone.0155103.s001.docx]

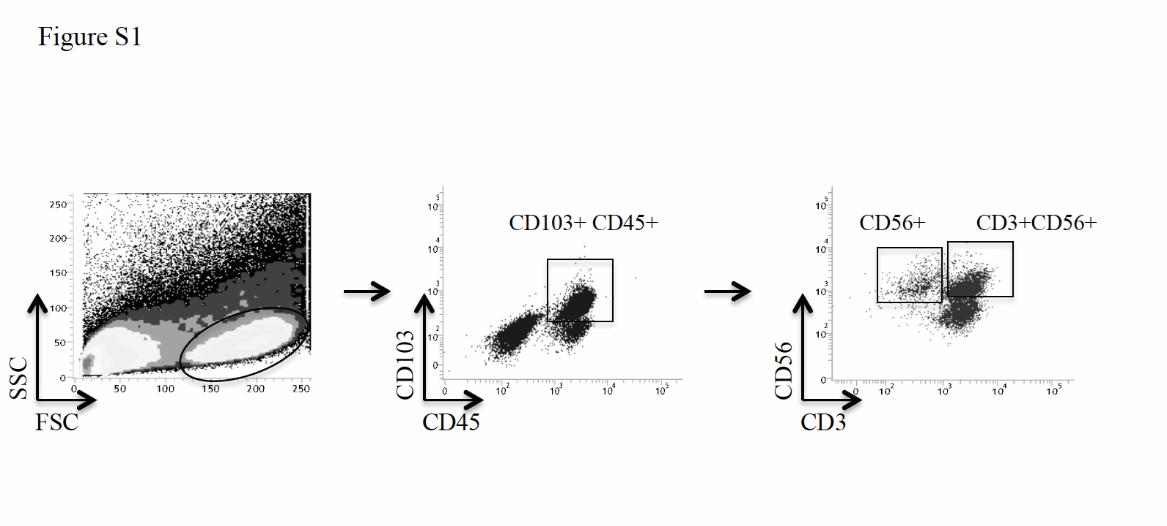


Figure S1. Gating strategy for representative dot plots of NK and NKT cells. Intraepithelial cells were gated on the lymphocytic area in the FSC/SSC plot, then on the CD45+ CD103+ population and finally on the CD3+CD56+ (NKT) cell and CD56+CD3- (NK) cell populations.
